# Occurrence of Bacterial Markers and Antibiotic Resistance Genes in Sub-Saharan Rivers Receiving Animal Farm Wastewaters

**DOI:** 10.1038/s41598-019-51421-4

**Published:** 2019-10-16

**Authors:** Dhafer Mohammed M. Al Salah, Amandine Laffite, John Poté

**Affiliations:** 1 University of Geneva, Faculty of Sciences, Earth and Environmental Sciences, Institute F. A. Forel and Institute of Environmental Sciences, Bd Carl-Vogt 66, CH-1211 Geneva 4, Switzerland; 2 King Abdulaziz City for Science and Technology, Joint Centers of Excellence Program, Prince Turki the 1st st, Riyadh, 11442 Saudi Arabia

**Keywords:** Environmental sciences, Hydrology

## Abstract

Antibiotic resistant bacteria and genes which confer resistance to antibiotics from human/animal sources are currently considered a serious environmental and a public health concern. This problem is still little investigated in aquatic environment of developing countries according to the different climatic conditions. In this research, the total bacterial load, the abundance of relevant bacteria (*Escherichia coli (E*. *coli)*, *Enterococcus* (Ent), and *Pseudomonas*), and antibiotic resistance genes (ARGs: *bla*_OXA-48_, *bla*_CTX-M_, *sul1*, *sul2*, *sul3*, and *tet(B)*) were quantified using Quantitative Polymerase Chain Reaction (qPCR) in sediments from two rivers receiving animal farming wastewaters under tropical conditions in Kinshasa, capital city of the Democratic Republic of the Congo. Human and pig host-specific markers were exploited to examine the sources of contamination. The total bacterial load correlated with relevant bacteria and genes *bla*_OXA-48_, *sul3*, and *tet(B)* (P value < 0.01). *E*. *coli* strongly correlated with 16s rDNA, *Enterococcus*, *Pseudomonas* spp., *bla*_OXA-48_, *sul3*, and *tet(B)* (P value < 0.01) and with *bla*_CTX-M_, *sul1*, and *sul2* at a lower magnitude (P value < 0.05). The most abundant and most commonly detected ARGs were *sul1*, and *sul2*. Our findings confirmed at least two sources of contamination originating from pigs and anthropogenic activities and that animal farm wastewaters didn’t exclusively contribute to antibiotic resistance profile. Moreover, our analysis sheds the light on developing countries where less than adequate infrastructure or lack of it adds to the complexity of antibiotic resistance proliferation with potential risks to the human exposure and aquatic living organisms. This research presents useful tools for the evaluation of emerging microbial contaminants in aquatic ecosystems which can be applied in the similar environment.

## Introduction

The global consumption of antibiotics between 2000 and 2015 has increased by approximately by 69%; that’s more than 4% increase annually. The spike in the consumption rate is quite alarming and has to be addressed^1^. Some countries have achieved a restriction on the sales of antibiotic and limited it to prescriptions by medical professionals. However, the sales of antibiotics in the rest of the world remains unmonitored and effortlessly accessible. The overuse of antibiotics and their subsequent poorly managed release to the environment especially aquatic systems have been linked with the development of antibiotic resistant characteristics^2–5^.

Wastewater effluent and effluent from animal farming and slaughter houses, where livestock consume staggering amounts of antibiotics for growth promotion, remain the most serious sources of antibiotic resistant genes. Even when such effluent goes under treatment, there’s more than sufficient evidence that ARGs remain detectable^6–8^. ARGs were found to remain detectable via molecular methods in the effluents from urban and hospital wastewater treatment plants^7,9^. The release of such contamination into the environment presents a great risk to the public health^10–13^.

The study of faecal contamination and ARGs outside clinical settings has gained momentum in the scientific community over last number of decades^14–18^. The assessment of the propagation and persistence of ARGs amongst all bacteria in general and human and animal pathogens especially in the environment will provide control measures to limit the harm of such genetic materials^8,19–22^. Waterbodies receiving typically wastewater contain a relatively high content of metals and provide a suitable environment for the selective pressure for antibiotic resistance and horizontal gene transfer (HGT) especially in warmer climates^23^. The occurrence of heavy metals in a given environment has shown to exert a selective pressure for antibiotic resistance bacteria. Moreover, low concentration of toxic metals have been shown to induce the conjugative transfer of ARGs^24,25^. However, sediments retain bacteria and antibiotics at a much higher scale than water and are less influenced by fluid dynamics. Therefore, sediments have been identified as reservoir of faecal indicator bacteria (FIB) and ARGs^26–28^ in aquatic environment according to the different climatic conditions.

The untreated/partial treated wastewater treatment plants (WWTP), urban, hospital, animal farms and industrial effluent waters can be considered as the main sources contributing to the dissemination of emerging contaminants (such as heavy metals, pathogens, ARGs, nutrients) into the aquatic environment. In developing countries, little information is available on the assessment of these contaminants in river receiving systems and few studies have been conducted to evaluate the effects of untreated hospital and urban effluent waters under tropical conditions including previous publications from our lab^23,29^. Moreover, studies on the contribution of animal farming to the proliferation of pathogens, ARGs in Sub-Saharan African rivers are quite limited. Consequently, in this study, we aimed to investigate the occurrence of ARGs and relevant bacteria marker genes in two different river systems in Kinshasa, Democratic Republic of Congo which are receiving effluent from animal farms to assess the contribution of the farms in the dissemination of antibiotic resistance. Also, we confirmed the source of contamination via detection of host-specific genetic markers. The assessment is based on Quantitative Polymerase Chain Reaction (qPCR) of ARGs (*bla*_OXA-48_, *bla*_CTX-M_, *Sul1*, *Sul2*, *Sul3*, and *tet(B)*) and relevant bacteria marker genes (*Escherichia coli*, *Enterococcus*, and *Pseudomonas* spp.) and PCR of host-specific markers (humans and pigs). To the best of our knowledge, this is the first study on the impact of animal farming on the propagation of ARGs and relevant bacteria in a central African region and specifically in the city of Kinshasa, the capital of the Democratic Republic of the Congo.

## Materials and Methods

### Sampling sites

**Figure 1:**
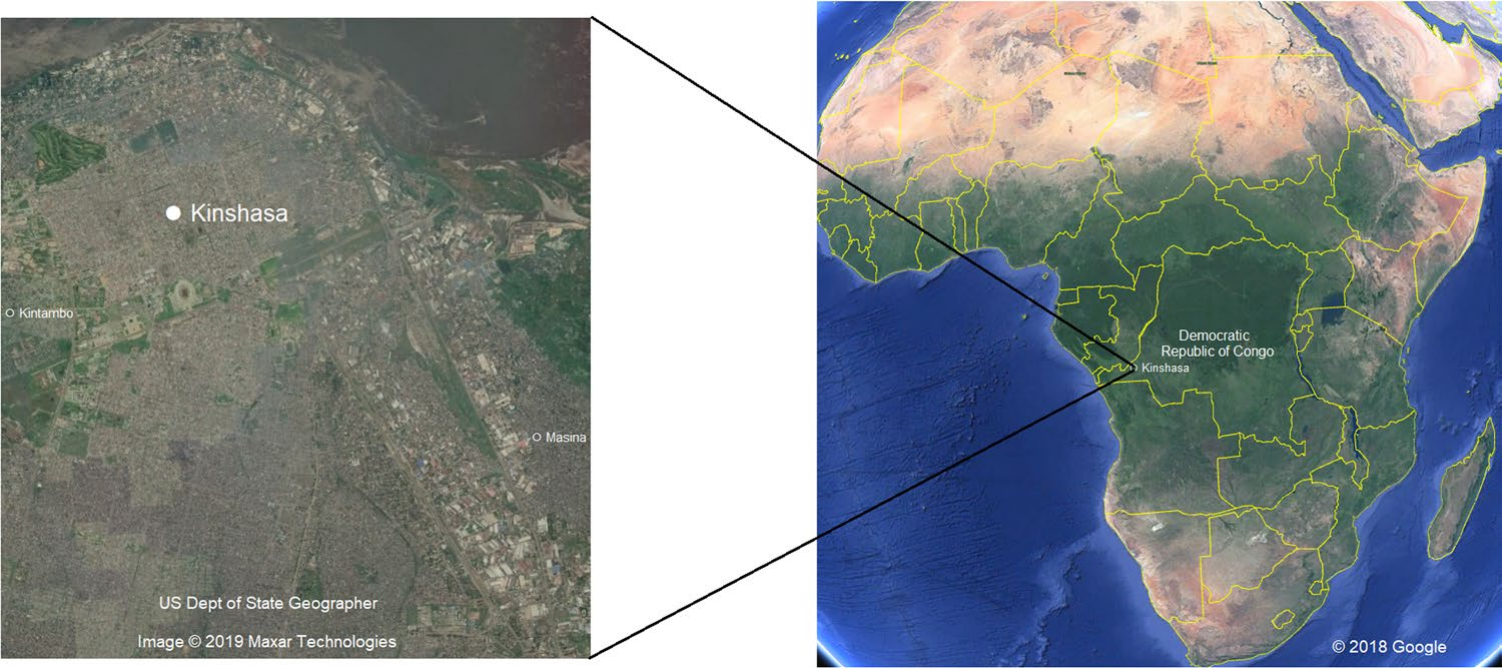
Map of the sampling location in Kinshasa and its location in relation to Democratic Republic of Congo and in relation to Africa (Adapted from Google Earth).

Two urban rivers were selected in the municipalities of Masina and Kintambo in the capital city of Kinshasa (DR Congo) to assess the impact of animal farming on the dissemination of ARGs (Fig. 1). Kinshasa is around 9,965 km^2^ and home to more than 11 million inhabitant. Each year Kinshasa receives a significant amount of people displaced by conflict, which contributes to the uncontrollable growth of urbanization in this Sub-Saharan Capital. Infrastructure, sanitation, and drainage system suffer a heavy load due to this growth that’s unaccounted for. Sampling procedure was similar to our previous publications^8,23,29^. The sampling took place in October, 2018. The letters K and M will be used to refer to Kintambo and Masina respectively in the text and figures. In each urban river, sediment samples were taken directly from the animal farms outlets which grew some cattle but mainly pigs (K eff and M eff) (Table 1) as well as downstream (K down and M down) and upstream (K up and M up) to the effluent discharge. Additionally, sediment samples were collected from Lake Ma Vallée to serve as controls because the lake doesn’t receive any wastewater effluents of any kind. Samples subsequently are preserved in an ice box at 4 °C before being shipped to the Department F.-A. Forel for Environmental and Aquatic Sciences at the University of Geneva and analyzed within two weeks.

**Table 1:** Sampling site description.

Municipality	Site description	Site name
Kintambo	Effluent from animal farm	K eff
	100 m upstream	K up
	100 m downstream	K down
Masina	Effluent from animal farm	M eff
	100 m upstream	M up
	100 m downstream	M down
Lake Ma Vallée	North of the Lake	control
	South of the Lake	control

### DNA extraction

DNA was extracted from a mass of about 0.2 g of each of the samples according to the manufacturer’s protocol of PureLink Microbiome DNA purification kit (Invitrogen). The exact mass, from which the DNA was extracted, was used for a more precise mean of DNA quantification. Extracted DNA was kept at −20 C until needed for downstream applications. The extracted DNA quality was examined by spectrophotometer (UV/VIS Lambda 365, PerkinElmer, Massachusetts, USA) using the 260/280 ratio which ranged between 1.74 and 1.92. The extraction volume was 50 μL and the concentrations ranged between 13.16 ng μL^−1^ and 54.66 ng μL^−1^.

### Faecal source tracking

The presence of host-specific markers of interest within the extracted genomic DNA was investigated using Polymerase Chain Reaction (PCR) with specific primer pairs for each of the human and pig markers (Table 2). Each PCR reaction was done in 50 µL volume which contains 5 µL of 10X DreamTaq buffer, 5 µL of 2 mM dNTP mix, 2.5 µl of 10 mM of each primer, 30 µL of molecular grade water, 0.25 µL of DreamTaq polymerase (5U/µL), and 5 µL of genomic DNA extracts. Biometra TOne thermocycler (Labgene) was used to perform an initial polymerase activation stage of 3 minutes followed by thirty cycles of 95 °C for 30 s, appropriate annealing temperature (Tm) for 30 s, and 72 °C for 1 min and a final 5 min extension at 72 °C. The PCR products were subsequently analyzed via 1.5% agarose gel electrophoresis. Samples with clear bands and a corresponding length to the marker of interest were considered positive.

**Table 2:** PCR Primer Pairs for ARGs screening and source tracking.

Target Gene	Primer	Sequence	Size of Target	Tm (°C)	Reference
Human-Specific bacteroidales	HF134	GCCGTCTACTCTTGGCC	591	49	^15^
	Bac708R	CAATCGGAGTTCTTCGTG			^14^
Pig-specific bacteriodales	PF163F	GCGGATTAATACCGTATGA	559	47	^51^
	Bac708R	CAATCGGAGTTCTTCGTG			^14^

### Quantification of ARGs and relevant bacteria markers

Primer pairs targeting different and specific genes for the qPCR have different efficiencies of amplification and varying annealing temperatures (Table 3). The quantification of the ARGs and the 16S rDNA was performed using SensiFAST SYBR No-Rox kit in an Eco qPCR System (Illumina) with a mix of 2 µL of 2X SensiFAST SYBR No-Rox mix, final concentration of 0.4 µM of primers, 1 µL of extracted DNA and molecular grade water up to 5 µL. The following cycling conditions were applied: 95 °C of polymerase activation for 3 min, followed by 40 cycles of denaturation at 95 °C for 5 s, optimal annealing temperature for 10 s, and extension at 72 °C for 10 s, and a final melting curve analysis. The interpretation of the qPCR results has been described in our previous publications^8,29^. In brief, six different dilutions of positive controls of known concentrations were used to construct the standard curve. Any samples with Ct value higher than or equal to negative control or the most diluted concentration of the standard curve were considered below the detection limit.

**Table 3:** Quantitative PCR Primer Pairs for the Quantification of Genes of Interest.

Target Gene	Primer	Sequence	Size of Target	Tm	R^2^	Amplification efficiency (%)	Reference
16s	338 F	ACTCCTACGGGAGGCAGCAG	197	62	0.99	102	^52^
	518 R	ATTACCGCGGCTGCTGG					
*E. coli* (UidA)	Uida 405 F	CAACGAACTGAACTGGCAGA	121	60	0.97	108	^53^
	Uida 405 R	CATTACGCTGCGATGGAT					
Pseudomonas spp.	Pse435F	ACTTTAAGTTGGGAGGAAGGG	251	62	0.99	94	^54^
	Pse686R	ACACAGGAAATTCCACCACCC					
Enterococcus (Ent)	Ent376F	GGACGMAAGTCTGACCGA	220	62	0.99	92	^55^
	Ent578R	TTAAGAAACCGCCTGCGC					
Sul 1	SulI-F	CGCACCGGAAACATCGCTGCAC	163	65	0.99	98	^56^
	SulI-R	TGAAGTTCCGCCGCAAGGCTCG					
Sul 2	SulII-F	CTCAATGATATTCGCGGTTTYCC	245	62	0.96	93	^57^
	SulII-R	AAAAACCCCATGCCGGGRTC					
Sul 3	sul3-F	GAGCAAGATTTTTGGAATCG	128	60	0.99	109	^56^
	sul3-R	CTAACCTAGGGCTTTGGA					
Tet (B)	tetB F	TACGTGAATTTATTGCTTCGG	206	60	0.97	96	^58^
	tetB R	ATACAGCATCCAAAGCGCAC					
Bla_**OXA-48**_	BlaOXA F	GCGTGGTTAAGGATGAACAC	438	61	0.99	98	^59^
	BlaOXA R	CATCAAGTTCAACCCAACCG					
Bla_**CTX-M**_	blaCTX-M F	ATTCCRGGCGAYCCGCGTGATACC	227	65	0.99	92	^60^
	blaCTX-M R	ACCGCGATATCGTTGGTGGTGCCAT					

### Statistical analysis

The R software version (3.5.2) was utilized to generate Principle Component Analysis (PCA) by inputting the absolute copy numbers of investigated markers and genes per gram of dry sediment^30^. The R package (Ade4) was used to generate the statistical data. The copy numbers were scaled to standardize the variance of the variables prior to the PCA analysis^31^. Also, the R software was utilized to produce Pearson Correlations Matrix at 95% confidence level.

## Results and Discussion

### PCR assays for source tracking

Faecal contamination originating from pig is occurring in all studied sites except for the samples from Lake Ma Vallée, the control site (Table 4). In the river from Masina, all sites were positive for contamination originating from pig feces; also, anthropogenic activities were detected upstream and downstream but not in the animal farm effluent. Similarly, in Kintambo, all sites were contaminated by pigs waste and contamination from human origins occurs upstream and downstream but not in the effluent from animal farming. Downstream and upstream of the effluents are affected by anthropogenic activities, which isn’t surprising due to the uncontrolled urbanization of Kinshasa and the unmanaged waste release. The control site from Lake Ma Vallée were negative for human and pig contamination. To summarize, all the sampling sites with the exception of Lake Ma Vallée were contaminated by bacteria originating from pigs.

**Table 4:** Occurrence/absence of ARGs.

Target host	Masina			Kintambo			Lake Ma Vallée
	D eff.	D up	D down	K eff.	K up	K down	Control
Humans	−	+	+	−	+	+	−
pigs	+	+	+	+	+	+	−

+detectable; −absent.

### Quantification of ARGs by quantitative PCR (qPCR)

**Figure 2:**
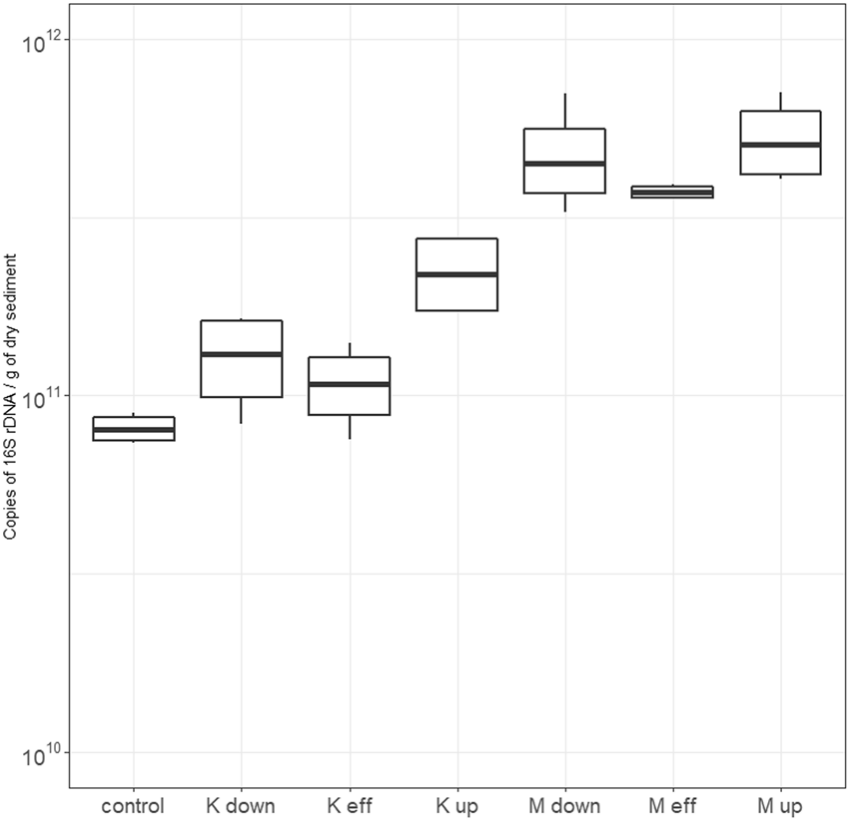
Absolute abundances of 16s rDNA per gram of dry sediment.

The absolute abundances of 16s rDNA are illustrated in (Fig. 2). The 16s rDNA abundances are expressed in log_10_ of copies per 1 g of dry sediment for a clearer presentation. The abundances of 16s rDNA varies significantly between Masina and Kintambo (p value < 0.01). The riverbed sediment from Masina housed significantly more 16s rDNA copies than Kintambo and the control site. The abundance ranged from its lowest at 8.13 × 10^10^ (control) to its highest in Masina (M up) upstream of the effluent at 5.35 × 10^11^ copies per gram of dry sediment, which isn’t surprising considering all the bacterial load brought in from urban wastewater and various anthropogenic activities compared to the control site. On average the sampling sites in Masina has 3.7 and 5.7 times the bacterial load as Kintambo and the control site respectively.

**Figure 3:**
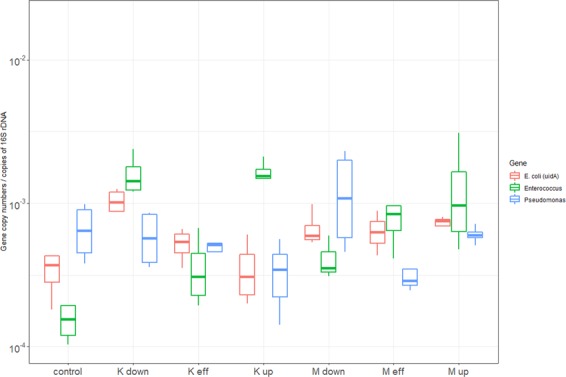
Relative abundances of bacterial markers genes expressed as log10 of copies per copy of 16s rDNA.

Normalized abundances of relevant bacteria marker genes is expressed in log_10_ total copy numbers per copies of 16s rDNA (Fig. 3). In a comparison between Masina, Kintambo, and the control sites, the average abundances of *E*. *coli*, *Enterococcus* and *Pseudomonas* in folds were 11.1 and 3.4, 30 and 1.9, and 5.8 and 5.4 respectively higher in Masina than the control site and Kintambo respectively. The relative abundance of *E*. *coli*, *Enterococcus* and *Pseudomonas* variations were significant between Masina, Kintambo and the control site (P value < 0.05); however, the variation upstream and downstream from the animal farm effluent weren’t which leads to the conclusion that the animal farming effluent didn’t contribute specifically to the degradation of the microbial quality of the rivers. On a different note, *E*. *coli* and *Enterococcus* relative abundances are higher in Masina and Kintambo than of the control site which concludes that both municipalities are contributing to the degradation of the microbial quality of the surface water. Masina district is a home to about four times the population of Kintambo which could attribute the higher copy numbers of all investigated markers and genes^32^. The contribution of wastewater investigated in this study site isn’t distinguishable when comparing downstream and upstream which could be attributed to a number of factors such as inadequate infrastructure and furrow release of wastewater. However, in the developed world where infrastructure is integral part of cities and even towns, the contribution of wastewater is noticeable and significant^9^.

Sulfonamides, and tetracycline resistance genes were selected for this study because of the usage of their perspective antibiotics in animal farming worldwide. Both tetracycline and sulfonamides are heavily used in the veterinary medicine to treat microbial infections and also as feed additives at lower concentrations to promote growth in livestock^33,34^. For instance, in 2012, the annual consumption of tetracycline in the United States exceeded 5954 tons^35^. Also, betalactam resistant genes were selected because more than half of the antibiotic prescribed to humans are betalactams^36^.

**Figure 4:**
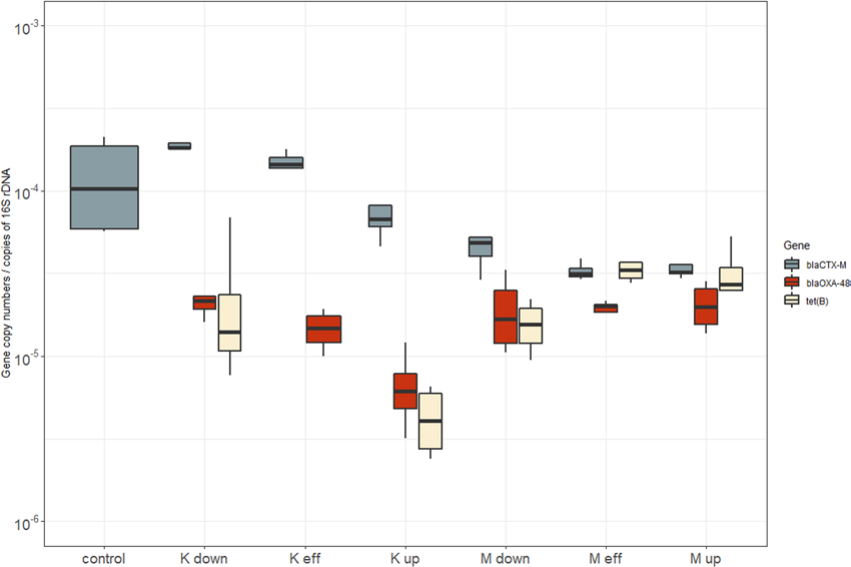
Relative abundances of betalactams and tet (B) expressed as log10 of copies per copy of 16s rDNA.

The relative abundances of *bla*_OXA-48_ and tet (B) are significantly different between Masina, Kintambo and the control site (p value < 0.05) but not *bla*_CTX-M_ (Fig. 4). *bla*_OXA-48_ and tet (B) aren’t detectable in the control site; however, *bla*_CTX-M_ was ubiquitous and detectable in all sites even in the control site that is expected as *bla*_CTX-M_ is considered one of the most widespread ARGs around the world^37^. Therefore, both municipalities are contributing to the spread of antibiotic resistant but not animal farming specifically. Moreover, *tet (B)* wasn’t detected in effluent from the animal farm in Kintambo.

**Figure 5:**
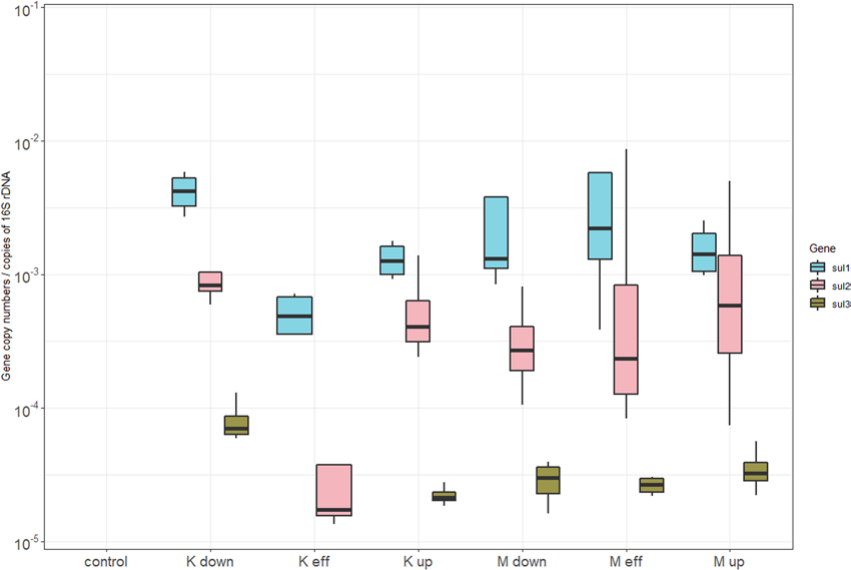
Relative abundances of sulfonamide resistance genes expressed as log10 of copies per copy of 16s rDNA.

The relative abundance of sulfonamide resistance gene (*sul1*, *sul2*, and *sul3*) is illustrated in (Fig. 5). The abundance in all the sites of *sul1* was the highest followed by *sul2* and finally by *sul3*. *Sul1* and *sul2* weren’t significantly different between Masina and Kintambo. However, all the sulfonamide resistance genes weren’t detectable in the control sites. This means that again that both municipalities are contributing to the spread of ARGs.

*Sul1* and *sul2* are the most abundant ARGs reported in this study which could be attributed to lack of infrastructure in the study sites and the overwhelming agricultural runoff from farms using manure as demonstrated in several studies performed in similar environment; e.g., in a study^38^, soils were supplied with antibiotic-free manure and with manure containing sulfonamides (10 or 100 ppm sulfadiazine in soil); Copy number of *sul1* and *sul2* increased with the application of the manure and increased even higher with the addition of sulfadiazine compare to the control soil. In a similar study conducted in China^39^, soils receiving compost and manure from pig farms showed a median ARGs enrichment of 192 times that of the control soils. In Canada and the United States^40^, research samples were soils receiving compost and manure from antibiotic-free animal farms and farms using subtherapeutic quantities of antibiotics; tetracycline resistance genes were only detected in isolates from farms using antibiotics for growth promotion.

Sediments are known to provide safe haven for bacteria by providing nutrient essential for growth and also provides a protection from degradation by sunlight^41,42^. The persistence of bacteria accompanied by Horizontal Gene Transfer (HGT) leads to the enrichment and proliferation of ARGs^43–45^. All these factors present a risk to the public health and further degradation of surface water quality.

### Statistical analysis

**Figure 6:**
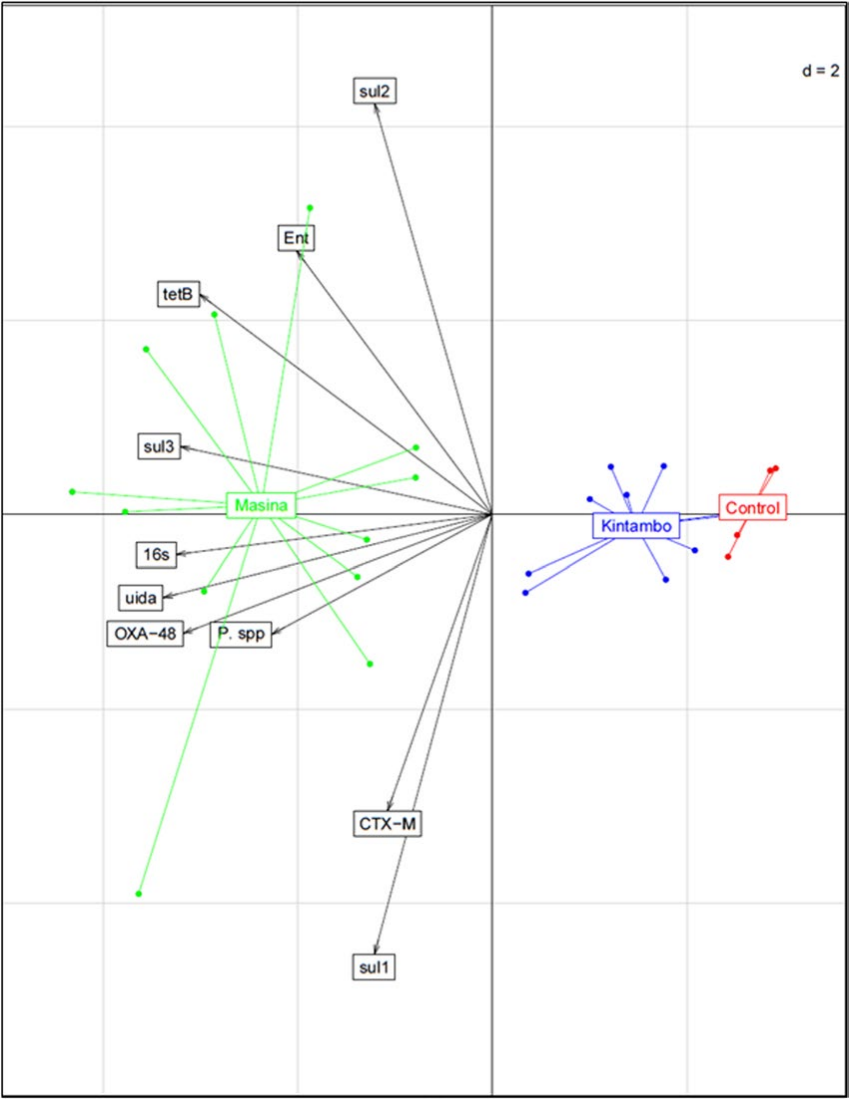
Principle Component and Cluster Analysis based on absolute abundances of 16s rDNA, bacterial markers, and ARGs per gram of dry sediments on correlation bipilot of the variables used for the analysis.

The data from the absolute abundances of the 16s rDNA, relevant bacteria markers, and ARGs per gram of dry sediments was processed to perform a principle component analysis (Fig. 6). The data was scaled to standardize the variance amongst the variables to eliminate the dominance of certain variables e.g. 16s rDNA in this case. The Principle component analysis is utilized in this study to illustrate the resemblance of bacterial composition in the study sites. The first principle accounted for 52.2% of the total variance and the second principle component accounted for 14.1%, which make a grand total of 66.3%. The correlation between the genes used in this study is quite clear from (Fig. 6) with a range of magnitudes. According to the analysis of variance (ANOVA), there wasn’t a significant different within groups. Therefore, the PCA and cluster analysis was utilized to illustrate between group analysis (BGA) and not within group analysis (WGA). Each municipality is forming its own cluster suggesting that Masina, Kintambo and the control site are distinctly different from one another. However, Kintambo and the control site clusters are closer to each other, which means that Kintambo is less contaminated than Masina which further backs up our earlier findings from the ARGs abundance comparisons.

Also, a Pearson correlation matrix was constructed to demonstrate the linear relationship between all the variables and its degree in a form of P value (Table 5). 16s rDNA is positively correlated with *E*. *coli*, *Pseudomonas*, *Enterococcus*, *bla*_OXA-48_, *sul3*, and *tet (B)* at a significance level of 0.01 and coefficients between 0.45 and 0.89. Moreover, *E*. *coli* is positively correlated with all the genes at the 0.01 significance level with the exception of *bla*_CTX-M_, *sul1* and *sul2* at the 0.05 level. *Enterococcus* and *Pseudomonas* don’t correlate with each other; however, they share the same correlation profile with all the other genes with only a single difference. *Enterococcus* and *Pseudomonas* positively correlate 16s rDNA, *E*. *coli*, *sul3*, and *tet(B)* at the 0.01 level and with *bla*_OXA,_ 0.05 and 0.01 level for *Enterococcus* and *Pseudomonas* respectively. In the next part of this paragraph, only the relationship between ARGs will be discussed to avoid repetition. *Bla*_OXA-48_ correlates with *sul1*, *sul3*, and *tet(B)* at the 0.01 level and *bla*_CTX-M_ only correlates with one other ARG which is *sul3*. *Tet (B)* correlates with all the genes are at the 0.01 level with the exception of *bla*_CTX-M_ and *sul1*. The persistence of ARGs has been long linked to the occurrence of faecal indicator bacteria e.g. *E*. *coli* and *Enterococcus*^46,47^. Also, other studies showed that the tropical climate such as our study sites further aids the efficiency of HGT which ultimately leads to the persistence and propagation of ARGs which represents a major public health issue^48–50^. The strong correlation of ARGs with *E*. *coli* support the fecal origin of these genes.

**Table 5:** Correlation Matrix among ARGS and bacterial population genes.

Correlations										
	16s	*E. coli*	Pseudomonas	Enterococcus	Bla_OXA-48_	Bla_CTX-M_	sul1	sul2	sul3	Tet(B)
16s	1	0.892**	0.644**	0.445**	0.876**	0,195	0,302	0,218	0.749**	0.775**
*E. coli*		1	0.551**	0.469**	0.927**	0.383*	0.429*	0.325*	0.827**	0.756**
Pseudomonas			1	0,179	0.451**	0,229	0,152	0,044	0.598**	0.475**
Enterococcus				1	0.369*	0,143	−0,153	0,114	0.717**	0.493**
Bla_OXA-48_					1	0,252	0.511**	0,276	0.719**	0.712**
Bla_CTX-M_						1	0,069	−0,137	0.329*	0,013
sul1							1	−0,090	0,190	0,148
sul2								1	0,251	0.513**
sul3									1	0.778**
Tet(B)										1

**Correlation is significant at the 0.01 level.

*Correlation is significant at the 0.05 level.

## Conclusion

This research investigates the occurrence trends of relevant bacteria and ARGs in two different rivers receiving effluent waters from animal farming under tropical conditions. These rivers serve as a basic network for human and animal consumption as well as irrigation for fresh urban produces. The findings from this study demonstrated that such aquatic systems can act as a reservoir of indicator bacteria (*Escherichia coli*, *Enterococcus* and *Pseudomonas*), and antibiotic resistance genes (*bla*_OXA-48_, *bla*_CTX-M_, *sul1*, *sul2*, *sul3*, and *tet(B)*) which could pose a further potential threat to the environment and human health.

On the other hand, the presence of higher values of relevant bacteria and ARGs in sediment samples located upstream of studied sites indicates that the animal farming wastewaters are not the only source of deterioration of the quality of studied rivers by investigated indicator bacteria and ARGs. Additionally, the faecal source tracking shows a variety of contamination originating from both pigs and anthropogenic activities.

The study concludes that effluent from animal farms aren’t the exclusive contributors in the propagation and the spread of ARGs but also anthropogenic activities. The lack of infrastructure, the furrow release of untreated wastewater, the unmonitored urbanization of Kinshasa, open defecation, septic tanks, over-the-counter antibiotics consumption and absence of antibiotics use regulation in humans, animals as well as for other agricultural purposes are probable sources of deterioration of the quality of the rivers. This study provides significant information indicating that sediments from tropical river receiving system could act as a potential reservoir of bacterial populations from human and animal sources. Thus, we suggest a well-monitored plan for the reduction of antibiotic consumption in animal farming to relieve the pressure applied for the selection of antibiotic resistance. The treatment or at least the partial treatment of the effluent will decrease bacteria of faecal origin that contribute to the propagation and persistence of such genes. Also, the uncontrolled urbanization in the Sub-Saharan Capital Cities and the lack of adequate infrastructure contribute to the spread of ARGs and has to be addressed.

### Ethic statement

We confirm that the field studies and sampling did not involve misunderstanding. The funder has no role in study design, data collection and analysis, decision to publish, or preparation of the manuscript.

## Data Availability

The datasets generated or analyzed during the current study are available from the corresponding author on reasonable request.
